# Pediatrics severe low back pain by disc herniation: an uncommon entity

**DOI:** 10.1186/s12969-023-00942-4

**Published:** 2024-01-02

**Authors:** Wendlassida Joelle Stéphanie Tiendrebeogo/Zabsonre, Denlewende Sylvain Zabsonre, Fulgence Kabore, Abdoulaye Sanou, Yakouba Haro, Inoussa Zoungrana, Dieu-Donné Ouedraogo

**Affiliations:** 1 Rheumatology Department of Bogodogo, University Hospital of Ouagadougou, Karpala, Burkina Faso; 2 Neurosurgery Department of Yalgado, Ouedraogo University Hospital of Ouagadougou, Karpala, Burkina Faso

**Keywords:** Low back pain, Disc herniation, Pediatrics, Discolysis

## Abstract

**Background:**

Common severe back pain due to disc herniation is rare in the paediatric population which involves children under eighteen years of age. Paediatric lumbar disc herniation (LDH) cannot be considered the same disease as in adults, as it has potentially different natural and clinical backgrounds. The treatment of pediatric LDH is the other particularity of this condition. Indeed, in children, delaying surgery for a conservative treatment is justified. We report 3 cases treated in 8 years.

**Case presentation:**

Three patients, two of whom were 14 years of age and one 17 years of age, were admitted for L5 or S1 lumbosciatica. A CT scan showed a lumbar disc herniation L5S1 associated with bi-isthmic lysis (and a transitional abnormality in 1 case or spina bifida occulta in 1 other case). The last patient had an magnetic resonance imaging (MRI) that showed a herniated L4L5 disc. The diagnosis of low back pain disc herniation was retained in two patients and that of disabling low back disc in one patient. Percutaneous discolysis in the two hyperalgesic cases and epidural corticosteroid infiltration in the disabling case were effective on lumbosciatica.

**Conclusion:**

Paediatric common lomw back pain caused by a disc herniation with a hyperalgic or disabling character posed a therapeutic problem which were solved by the invasive approaches that must be given priority nowadays with children.

## Background

Common low back pain due to disc herniation is rare in the paediatric population. In practice, the paediatric population consists of children under 18 years of age. In this population, lumbar disc herniation (LDH) is a rare cause of morbidity that can lead to school disruptions and non-participation in social and sports activities. Indeed, it causes pain that evolves intermittently and lasts in time [1]. The diagnosis of this herniated disc poses few problems with CT scans or magnetic resonance imaging as with herniated discs in adults. However, paediatric LDH cannot be considered the same disease as in adults, as it has potentially different natural and clinical history [2, 3]. The treatment of pediatric HDL is the other particularity of this condition. Indeed, in children, delaying surgery for conservative treatment is justified, but for how long? In case of surgical indication, the classic open discectomy widely used in previous decades has been increasingly replaced by modern minimally invasive techniques that are increasingly adopted in pediatric surgery. They have the advantage of reducing connective tissue damage while promoting clinical improvement [3, 4]. We report three paediatric cases of common low back pain due to disc herniation (including 2 severe cases and 1 disabling case) in black african children in a country with limited resources.

## Case presentation

### Case 1

A 14-year-old girl, student who commutes to school daily by bicycle, was admitted in rheumatologic department for severe left S1 lumbosciatica that had been evolving for about three weeks after a sudden onset following efforts to ride her bike. This pain was resistant to the usual analgesics. The patient did not report any particular medical history apart from low back pain occurring intermittently after a certain bicycle ride. At admission, the patient rated her pain on the Visual Analog Scale (VAS) as 10/10. The physical examination noted that the bedridden patient holding an analgesic posture when lying down because standing and walking were impossible. Lumbar spinal syndrome (pain on palpation of the lumbar spinous processes, contracture of the paravertebral muscles) and a positive Lasegue sign at 15 degrees were noted. There were no neurological deficiency.

**Fig. 1:**
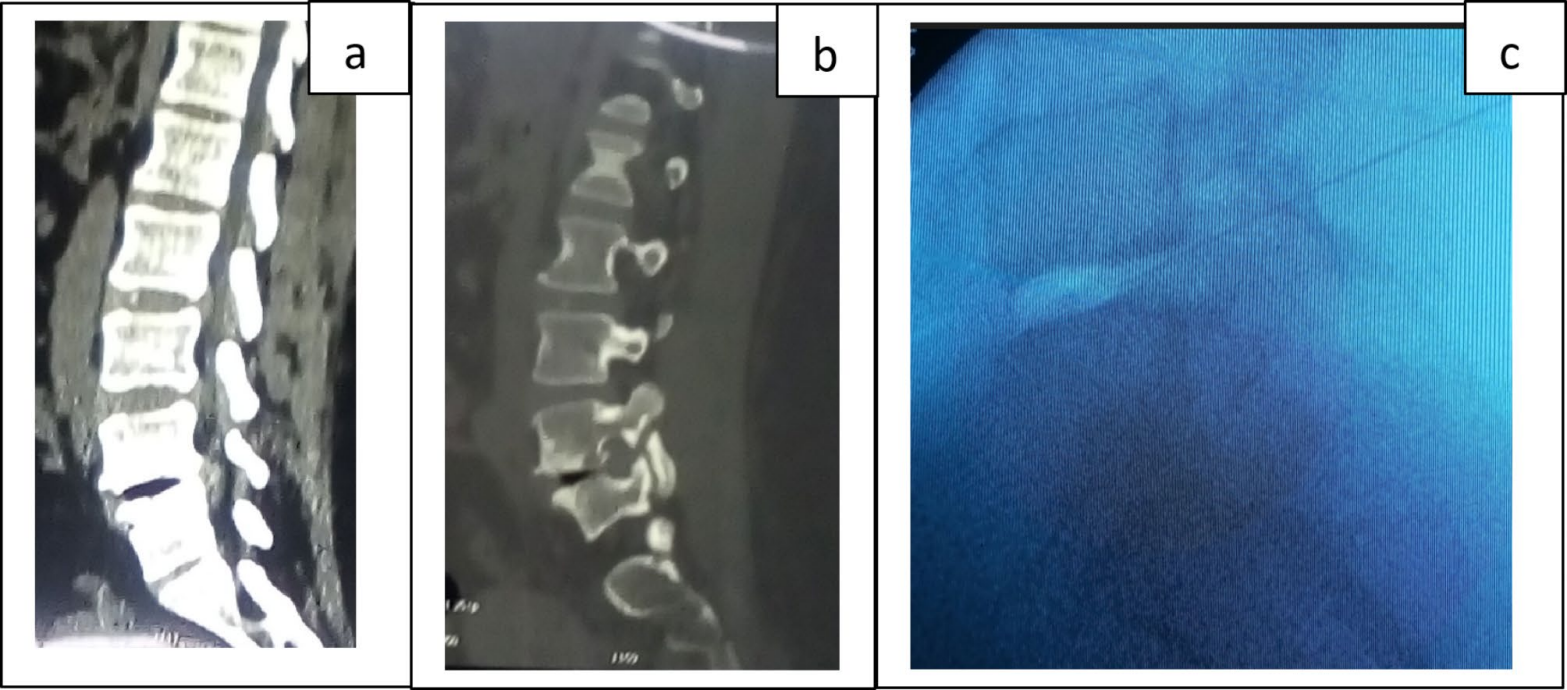
CT and per discolysis fluoroscopy images of the patient of the observation 1. CT sagittal reconstruction, parenchymal window, passing through the spinal canal (**a**) showing a disc vacuum L5S1 and a herniated L5S1 on an S1 lumbalization; CT in sagittal reconstruction, bony window passing through the posterior articular (**b**) visualizing an L5S1 disc vacuum and an undisplaced isthmic lysis of L5; image of fluoroscopy during the discolysis procedure (**c**) objectifying the needle of the discolysis implanted in L5S1 and the opacity of the ozone that was injected

Blood count, C reactive protein, blood ionogram, and serum creatinine were normal. Computed tomography (CT) of the lumbar spine showed non-displaced bi-isthmic lysis of L5 with a disc void and a compressive L5S1 disc herniation. There was also a transitional anomaly with the type of lumbalization of S1 (Fig. 1a and b). The diagnosis of common back pain by disc herniation on an isthmic lysis and a transitional vertebra was retained. The patient was hospitalized. Drug injectable therapy with paracetamol combined with morphine, thiocolchicoside and methyl prednisolone has been initiated. Epidural corticosteroid infiltration was also performed on the fourth day of hospitalization. Faced with the persistence of lumbosciatica which still remained very intense after 08 days of drug treatment above, the diagnosis of severe back pain was arrested and the patient was referred to neurosurgery where percutaneous surgery (ozone L5S1 discolysis) was performed (Fig. 1c). The postoperative period was simple and marked by a progressive improvement in pain. The patient was discharged from hospital on the second day of discolysis on oral analgesics (paracetamol and tramadol) and hygienic dietary measures. The VAS at the outlet was 1/10. The patient was seen again one week after discharge; she reported no spontaneous pain (but bearable pain was noted after some physical activity). She had been back to school for 2 days. Continued compliance with spinal hygiene measures, analgesics (paracetamol) in case of pain were prescribed. The patient was seen again one month and three months later. The evolution was good without any treatment.

### Case 2

**Fig. 2:**
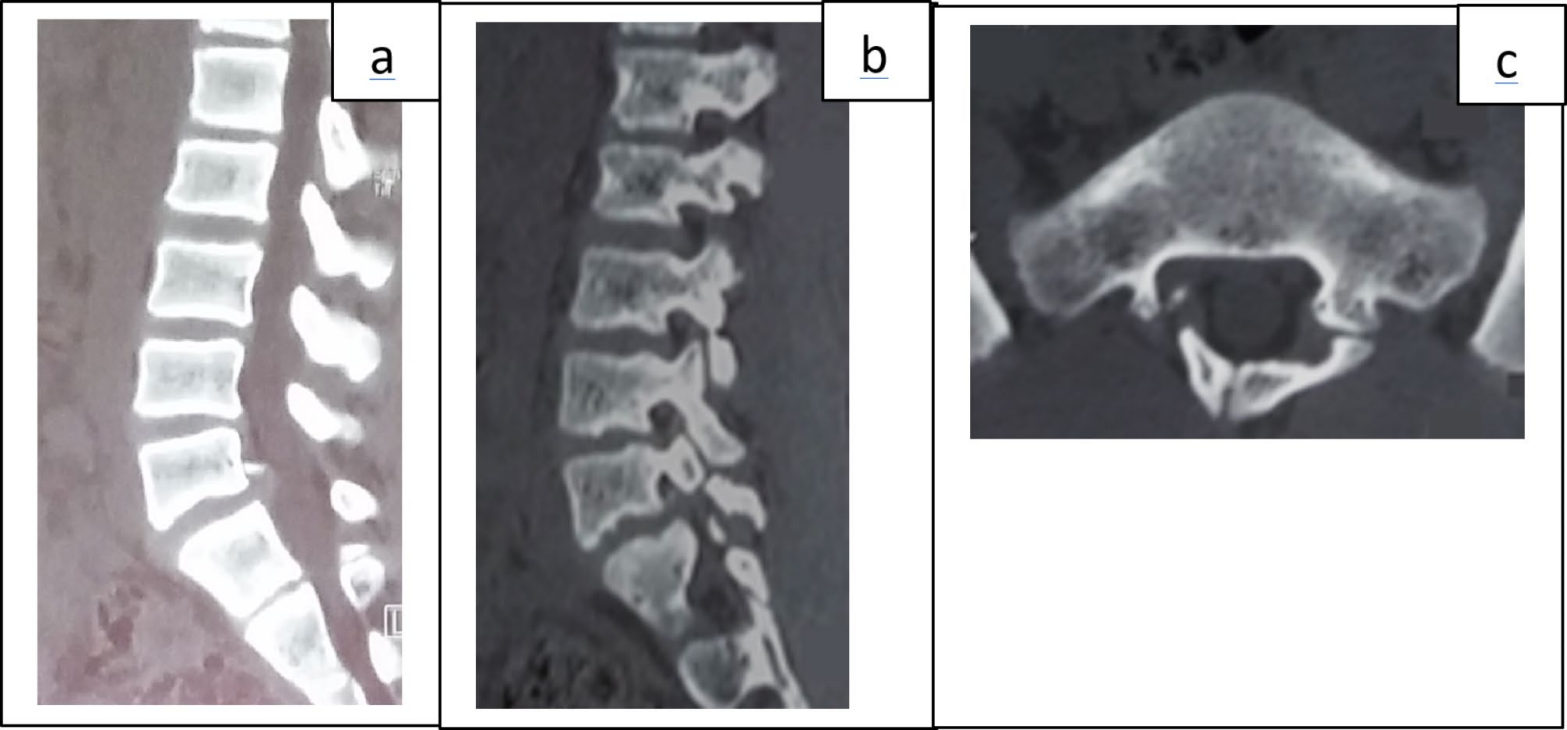
CT images of the patient of the observation 2. CT in sagittal reconstruction, parenchymal window, passing through the spinal canal (**a**) showing a calcified L5S1 disc herniation in its upper part; CT in sagittal reconstruction, bony window, passing through the posterior articular (**b**) visualizing an undisplaced isthmic lysis of L5; CT in axial section, bony window passing through S1 showing an anomaly of closure of the posterior arch of S1 (spina bifida occulta) (**c**)

A 14-year-old patient, was admitted in rheumatology department for very intense bilateral lumbosciatica that had been progressing for about one month, with a sudden onset following a squatting position. This pain was resistant to the usual analgesics. The patient did not report any particular disease history apart from intermittent low back pain. At admission, the patient rated his pain on the visual analogue scale (VAS) at 9/10. Physical examination noted an analgesic posture in flexion of the spine. Standing and walking were very laborious in flexion of the spine. Lumbar spinal syndrome (pain on palpation of the lumbar spinous processes, contracture of the paravertebral muscles) and radicular syndrome (consisting of a sign of the doorbell in the face of L5S1, a positive Lasegue sign at 30 degrees) were noted. There were no neurological deficits. Blood count, C reactive protein, blood ionogram, and serum creatinine were normal. Computed tomography (CT) of the lumbar spine showed non-displaced bi-isthmic lysis of L5 with a partially calcified and compressive L5S1 disc herniation. There was also an abnormality in the closure of the posterior arch of S1 (spina bifida oculta) (Fig. 2). The diagnosis of common severe back pain by a disc herniation on isthmic lysis and spina bifida oculta was retained. The patient was hospitalized. Drug treatment with parenteral administration of paracetamol combined with tramadol, a muscle relaxant and methyl prednisolone has been initiated. Faced with the persistence of the lumbosciatica which still remained hyperalgic after one week of the above drug treatment, percutaneous surgery was performed (L5S1 ozone discolysis). The postoperative period was simple and marked by a progressive improvement in pain. The patient was discharged from hospital on the second day of discolysis on analgesics and hygienic dietary measures. The VAS was 0/10. The patient was seen again one week after discharge, she reported no pain. Continued compliance with spinal hygiene measures, oral analgesics (paracetamol) in case of pain were prescribed. The course was favourable at one month and then at three months without treatment.

### Case 3

A 17-year-old patient, was admitted for disabling bilateral L5 lumbosciatica type, predominantly on the right who had been progressing for about 1 month following the lifting of a load. This pain responded to the usual analgesics and then resumed at the slightest activity disabling the patient. The patient did not report any particular medical history. At admission, the patient rated his pain on the visual analog scale (VAS) at 6/10 at rest and 9/10 at the slightest movement.

**Fig. 3:**
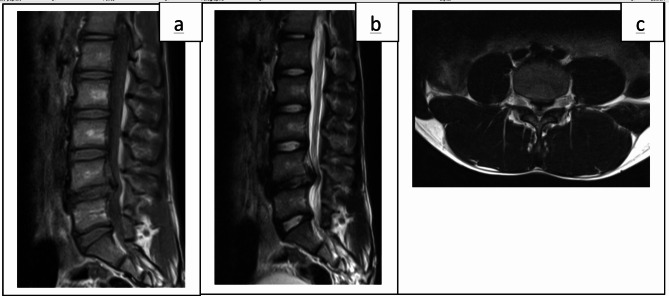
MRI images of the patient of the observation 3. Sagittal section in weighted sequence T1 (**a**); T2 (**b**); axial section in T2-weighted sequence (**c**) to highlight a compressive posteromedial L4L5 disc herniation; the disc L4L5 being always well hydrated

Physical examination noted an analgesic posture in flexion of the spine. Standing and walking were possible with slight flexion of the spine. A lumbar spinal syndrome (consisting of spinal stiffness with a distance from the toes to the ground at 60 centimeters, pain on palpation of the lumbar spinous processes at L5, contracture of the paravertebral muscles) and radicular syndrome (made up of a bilateral bell sign at L4L5, a positive Lasegue sign at 50 degrees, a right L5 paresis at 4/5) were noted. There were no genito-sphincter disorders Blood count, C reactive protein, blood ionogram, and serum creatinine were normal. Magnetic resonance imaging (MRI) revealed a compressive posterior medial L4L5 disc herniation on the dural sheath. The L4L5 disc was still well hydrated (Fig. 3). The diagnosis of disabling common severe low back pain due to a herniated disc was retained. The patient was hospitalized. Parenteral treatment consisting of paracetamol combined with tramadol, thiocolchicoside and methyl prednisolone has been initiated. Epidural corticosteroid infiltration was also performed by the rheumatology team. The pain gradually improved and after 5 days of treatment the patient was asymptomatic, the VAS at 0/10 and the physical examination normal. This had allowed him to leave the hospital on the fifth day of hospitalization for his home on oral analgesics (paracetamol) to be taken in case of pain and hygienic dietary measures.

## Discussion

Low back pain due to a disc herniation is a rare condition in pediatrics. The first Indonesian case was described in 2022 in a 15-year-old patient who had been experiencing deteriorating back pain and left leg pain for more than 4 years [5]. Also in 2022, two Chinese cases were published [6]. Other authors had described 12 cases in 5 years [7], 70 cases in 4 years [3]; 74 cases in 10 years [8]. Pediatric LDH rarely occurs in children younger than 12 years of age [2]. The mean age was 15.4 [9]; 12.6 years (11–16 years) [7]; 17.14 ± 2.15 [3]. The three cases we reviewed had some similarities. First, the damage was localized to a single disc in the lumbar spine; Then the radiculalgia was well systematized and a triggering factor was well identified. This could be explained by the fact that they are young rachis that are not degenerated as a whole. The associated vertebral abnormalities in 2 of our 3 patients with bi-isthmic lysis and/or spina bifida and/or transition vertebra could be considered as contributing factors to these disc lesions in children. Authors cited cystic fibrosis, trauma, strenuous athletic activity, facet joint asymmetries, and lumbosacral transition vertebrae as potential risk factors for pediatric LDH [2]. They point out that these cases usually present with a well-hydrated disc and are more often associated with an avulsion fracture of the annular epiphysis [2].

The paediatric lumbar disc herniation was located in the lumbar spine in all the series we consulted for this work. The L4L5 and L5S1 levels were the most affected. The hernia was located in L4L5 (52%) and L5S1 (41%) [2]. It sat at the L3L4 level in 3% of cases, L4L5 in 49% and L5S1 in 48% [8]. Other authors had an L5S1 level in all their two cases [6].

CT scan was sufficient for a positive diagnosis whenever it was requested as a first-line measure in our first 2 observations. MRI was requested as a first line intent for the patient who came from the province. This could be due to the fact that one wanted to take no risk of going to the diagnosis starting with a CT scan. Indeed, in the literature, MRI is the examination of first choice for the diagnosis of disc damage [3, 4]. In addition, it is a less invasive examination than CT and therefore more indicated for younger patients.

The treatment of low back pain by paediatric disc herniation is a real challenge when the lumbosciatica is hyperalgic or disabling and resistant to the usual analgesics, as was the case in our patients. Indeed, the classic indications for surgery in lumboradiculalgia due to herniated discs are hyperalgic radiculalgia, disabling radiculalgia and paralyzing radiculalgia. Thus our three observations constitute real surgical indications. The first two for hyperalgic low back pain and the third for disabling low back pain. However, the recurrent nature of the pain, even after surgery, makes it necessary to insist as much as possible on conservative treatment for four to six weeks, especially in children [3, 4]. In addition, in our first two cases there was bi-isthmic lysis. This would be indicative of bone synthesis in case of conventional open surgery [9]. However, in growing children, the set up of osteosynthesis equipment raises the difficult question of its removal. We were able to overcome this pain with percutaneous ozone surgery in both cases of bi-isthmic lysis. This percutaneous surgery is a less aggressive procedure, which better respects the anatomy with satisfactory results [4, 10]. In our last observation, epidural corticosteroid infiltration was effective. These treatments have allowed us to spare our patients a classic and serious open surgery, especially in children. In children, therefore, emphasis should be placed on the least aggressive procedures for open surgery whenever possible. Elsewhere, the results of managing paediatric LDH with minimally invasive methods were satisfactory. Thus, transforaminal percutaneous endoscopic discectomy had given excellent or good results in 91.6% and no patient had required a new surgery [7].

## Conclusion

Common low back pain by pediatric disc herniation with hyperalgesic or disabling character had a well-identified precipitating factor and a precise root path. CT was sufficient for diagnosis. It made it possible to objectify other vertebral disorders (bi-isthmic lysis, spina bifida occulta, transition vertebrae) associated with LDH. These associated lesions must have facilitated the LDH. Only one level was reached per patient. The L4L5 and L5S1 levels were the most affected. Patchy discolysis and epidural corticosteroid infiltration relieved the pain. Subsequently, good compliance with spinal hygiene measures made it possible to prolong this lull over time. In pediatric settings, conventional open surgery, once the most widely used, is now being abandoned in favour of modern minimally invasive techniques for the management of LDH.

## Data Availability

The datasets used and/or analysed during the current study are available from the corresponding author on reasonable request.

## List of abbreviations

LDH: lumbar disc herniation
CT: Computed tomography
VAS: Visual Analog Scale
MRI: Magnetic resonance imaging
